# Therapeutic Effects of 15 Hz Pulsed Electromagnetic Field on Diabetic Peripheral Neuropathy in Streptozotocin-Treated Rats

**DOI:** 10.1371/journal.pone.0061414

**Published:** 2013-04-18

**Authors:** Tao Lei, Da Jing, Kangning Xie, Maogang Jiang, Feijiang Li, Jing Cai, Xiaoming Wu, Chi Tang, Qiaoling Xu, Juan Liu, Wei Guo, Guanghao Shen, Erping Luo

**Affiliations:** 1 School of Biomedical Engineering, Fourth Military Medical University, Xi’an, China; 2 Department of Neurology, Xijing Hospital, Fourth Military Medical University, Xi’an, China; 3 School of Nursing, Fourth Military Medical University, Xi’an, China; National Research Council, Italy

## Abstract

Although numerous clinical studies have reported that pulsed electromagnetic fields (PEMF) have a neuroprotective role in patients with diabetic peripheral neuropathy (DPN), the application of PEMF for clinic is still controversial. The present study was designed to investigate whether PEMF has therapeutic potential in relieving peripheral neuropathic symptoms in streptozotocin (STZ)-induced diabetic rats. Adult male Sprague–Dawley rats were randomly divided into three weight-matched groups (eight in each group): the non-diabetic control group (Control), diabetes mellitus with 15 Hz PEMF exposure group (DM+PEMF) which were subjected to daily 8-h PEMF exposure for 7 weeks and diabetes mellitus with sham PEMF exposure group (DM). Signs and symptoms of DPN in STZ-treated rats were investigated by using behavioral assays. Meanwhile, ultrastructural examination and immunohistochemical study for vascular endothelial growth factor (VEGF) of sciatic nerve were also performed. During a 7-week experimental observation, we found that PEMF stimulation did not alter hyperglycemia and weight loss in STZ-treated rats with DPN. However, PEMF stimulation attenuated the development of the abnormalities observed in STZ-treated rats with DPN, which were demonstrated by increased hind paw withdrawal threshold to mechanical and thermal stimuli, slighter demyelination and axon enlargement and less VEGF immunostaining of sciatic nerve compared to those of the DM group. The current study demonstrates that treatment with PEMF might prevent the development of abnormalities observed in animal models for DPN. It is suggested that PEMF might have direct corrective effects on injured nerves and would be a potentially promising non-invasive therapeutic tool for the treatment of DPN.

## Introduction

Diabetic peripheral neuropathy (DPN) is generally considered to be one of the most common complications of diabetes mellitus, affecting both types of diabetes equally [1]–[3]. Studies suggest that about 30% of patients with diabetes mellitus are affected by DPN and 16–26% of diabetic patients experience chronic pain [4].

DPN is characterized by aberrant symptoms of stimulus-evoked pain including allodynia and hyperalgesia [5], and it often leads to mood and sleep disturbance, and thus can substantially impair the quality and expectancy of life [6], [7]. Therefore, it imposes a huge burden on both individuals and society, and represents a major public health problem. However, beyond the careful management of the diabetes itself via glycemic control and pain relief for neuropathy, no treatment for DPN exists [8], [9]. Potential toxicity, poor tolerability and ineffectiveness for some percent of diabetic patients are major disadvantages of the current therapeutic options. For this reason, there is a need to explore other non-pharmacological novel therapeutic modalities with efficacy and safety, particularly when diabetic patients require a combined treatment with an oral antidiabetic drug to prevent the development of DPN.

Numerous clinical studies have reported that pulsed electromagnetic fields (PEMF) are able to modify some parameters of nerve function in diabetic patients [10], [11], and a voluminous amount of literature has suggested that PEMF can stimulate nerve growth, regeneration, and functional recovery of nerves in cells in vitro or in animal models with nerve disease [12]–[16]. However, the application of PEMF for clinic is still controversial [17]. Therefore, more research is needed to confirm the therapeutic effects of PEMF on DPN and then to justify the applicability of PEMF for clinical practice. Since few studies have examined the effects of PEMF on neuropathy induced by diabetes mellitus in animals at present, this study aimed to test whether PEMF has therapeutic potential in relieving diabetes-induced neuropathy in animals.

Streptozotocin (STZ)-induced diabetic rat model has been used extensively as a model of DPN to demonstrate many abnormalities observed in patients with DPN and to assess the efficacies of potential therapeutic interventions [18]–[20]. Diabetic rats develop tactile allodynia and hyperalgesia to mechanical or thermal stimuli in the hind paws two or three weeks after STZ injection [5], [21], [22]. In the current study, we examined the effects of whole-body exposure to 15 Hz PEMF whose peak magnetic flux density (MFD) was approximately 1.6×10^−3^ T on improving signs and symptoms of DPN in STZ-treated rats by using behavioral assays. The PEFM was generated by a modified Helmholtz coils and the exposure duration was 8 hours everyday, 6 days a week for 7 weeks. Meanwhile, ultrastructural examination and immunohistochemical study for vascular endothelial growth factor (VEGF) of sciatic nerve were also performed seven weeks after PEMF stimulation. Moreover, the potential action mechanism of PEMF on DPN was preliminarily investigated.

## Methods

### Experimental Diabetes

Thirty adult male Sprague–Dawley rats, weighting 350±20 g, were provided by Animal Center of the Fourth Military Medical University and housed in a room (Animal Center of the Fourth Military Medical University, Xi’an, China) with controlled temperature (23±1°C), relative humidity (50∼60%), and alternately light-dark cycle (12 h/12 h), with access to standard pellet and clean water. Diabetes mellitus was induced by an intraperitoneal injection of STZ (Sigma Chemicals, St. Louis, MO, USA) at 45 mg/kg in freshly prepared 0.1 mM citrate buffer (pH 4.5) after an overnight fast [23]. Confirmation of hyperglycemia was made three days after STZ injection, and only STZ-treated rats whose glucose concentration of the tail venous blood measured by OneTouch SureStep Plus glucometer (Lifescan, Milpitas, CA, USA) was higher than 20 mM were considered as qualified diabetic models [24]. Six rats were excluded from the study after confirmation of success of diabetic models because of low blood glucose levels. The rest of rats were randomized into three weight-matched groups (eight in each group): the non-diabetic control group (Control), diabetes mellitus with sham PEMF exposure group (DM), diabetes mellitus with PEMF exposure group (DM+PEMF) which were subjected to whole-body exposure to PEMF 8 hours (09:00–17:00) everyday, 6 days a week for 7 weeks. Although the same PEMF apparatus was employed in DM group, the PEMF stimulation was not activated. PEMF stimulation was carried out the next day after confirmation of hyperglycemia. The current study was performed in adherence to the National Institutes of Health guidelines for the use of experimental animals, and all animal protocols were approved by the Committee for Ethical Use of Experimental Animals of the Fourth Military Medical University.

### PEMF Apparatus

**Figure 1:**
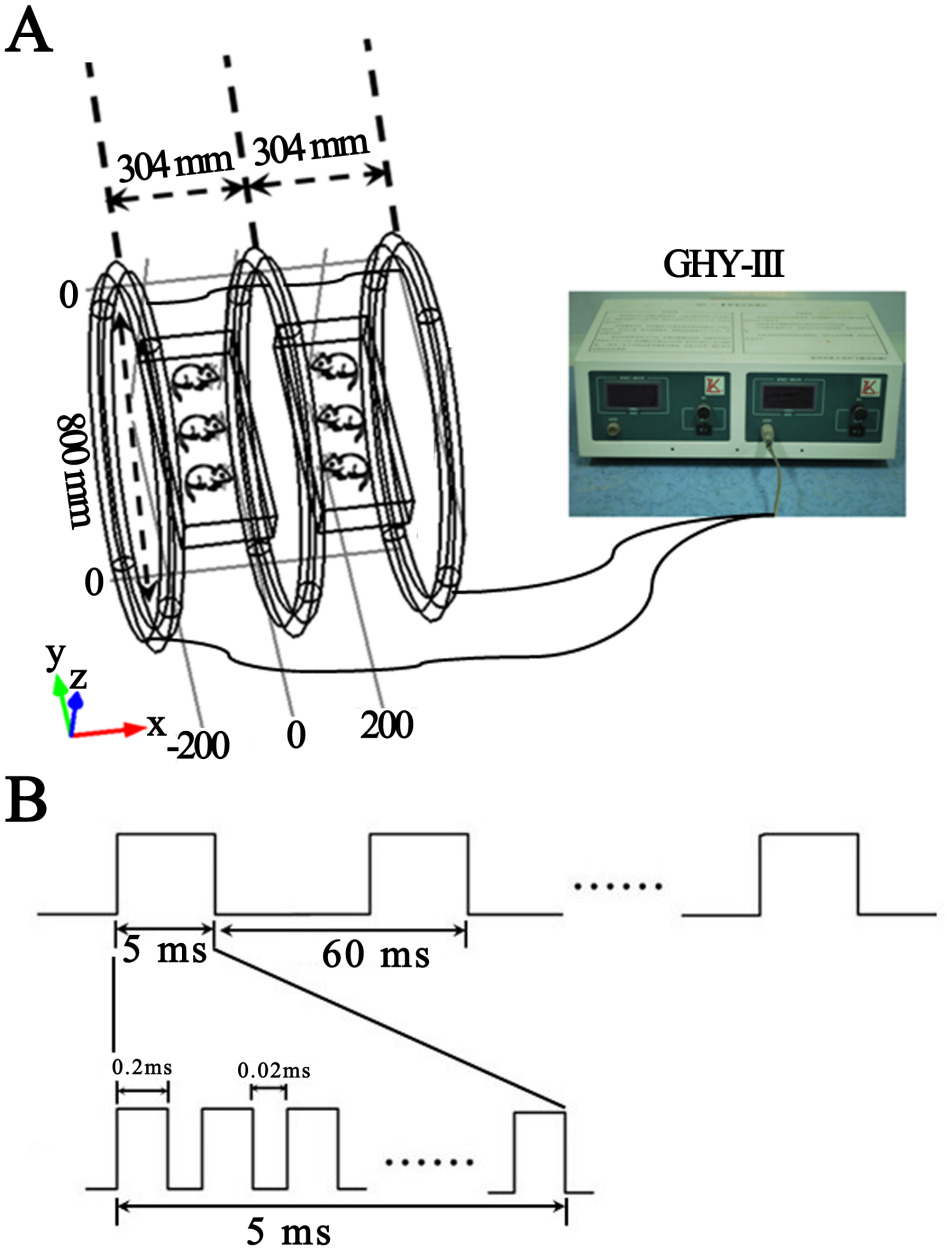
Schematic drawing of PEMF exposure system and PEMF pulse protocol. (**A**) Modified Helmholtz coils consisted of three identical coils with diameters of 800 mm which were in series connection and mounted coaxially at a distance of 304 mm apart. Two cubic plastic rat cages whose length was along O–Y direction (Origin is the center of the middle coil, O–X direction is the axial direction of the coil and the coordinate system meets right-hand rule) were put in the center of every two neighboring coils and cages were supported by stands to let the activities of rats restrict on the XY plane. The modified Helmholtz coils were wired to the GHY-III pulse generator. (**B**) The pulse stimulator (GHY-III) generated an open-circuit voltage waveform of PEMF with a repetitive burst frequency at 15 Hz (burst width, 5 ms; burst wait, 60 ms; pulse width, 0.2 ms; pulse wait, 0.02 ms; pulse rise and fall time: 0.3 µs, 2.0 µs).

Three identical coils with coil diameters of 800 mm constituted the PEMF stimulation apparatus (the modified Helmholtz coils). The coils were in series connection and placed coaxially with a distance of 304 mm apart (Fig. 1A). Each coil was made up of enameled coated copper wire with 0.8 mm diameter. The assembly of three identical coils significantly upgraded the uniformity of MFD by decreasing the deviation of the MFD between the central reference point (origin, center of the middle coil) and other areas in the magnetic field [25].

The MFD value along the O–X direction (axial direction of the coil, the coordinate system meets right-hand rule) is expressed as:
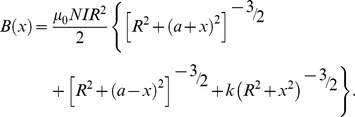



Where 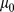 is the permeability of vacuum, *I* is the current through the coils, *R* is the radius of the coils, 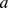 is the distance between the central coil and the outside coil, 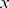 is the abscissa relative to origin, *N* is the number of turns of the outside coil, and *k*×*N* is the number of turns of the middle coil. By setting the parameters 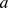 = 0.7601*R* and *k* = 0.5315, the second and fourth derivative of *B(x)* will become zero at the position of origin and then the maximum uniformity of the MFD will be obtained [25]. In the present study, we set the number of turns of the two outside coils as 500. Therefore, the number of turns of the central coil has been determined as 266. Besides, the distance between the central coil and the outside coil was approximately 304 mm. The modified Helmholtz coils were wired to a pulse generator (GHY-III, FMMU, Xi’an, China; China Patent no.ZL02224739.4) which produced a PEMF signal (Fig. 1A). The open-circuit voltage waveform of the PEFM consisted of a pulsed burst (burst width, 5 ms; burst wait, 60 ms; pulse width, 0.2 ms; pulse wait, 0.02 ms; pulse rise and fall time: 0.3 µs, 2.0 µs ) repeated at 15 Hz (Fig. 1B). The reason for selecting this particular waveform was that it had been proven to be effective in diabetes-induced diseases in our previous studies which were performed by our study group over a long period of time [26]–[30].

Two cubic plastic rat cages containing rats in PEFM group were put in the center of every two neighboring coils (the length of the cage was along O-Y direction) and cages were supported by stands to let the activities of rats restrict on the XY plane which had higher intensity and better uniformity of MFD (Fig. 1A). Moreover, whole body exposure to PEMF for rats was applied eight hours everyday. The distribution of the peak MFD was measured by using a Gaussmeter (Model 455 DMP Gaussmeter, Lake Shore Cryotronics, USA), and the measurement result was (1.6±0.1)×10^−3^ T (average ± standard deviation) in the exposure area (cage: 50 cm long, 20 cm wide and 15 cm high).

**Figure 2:**
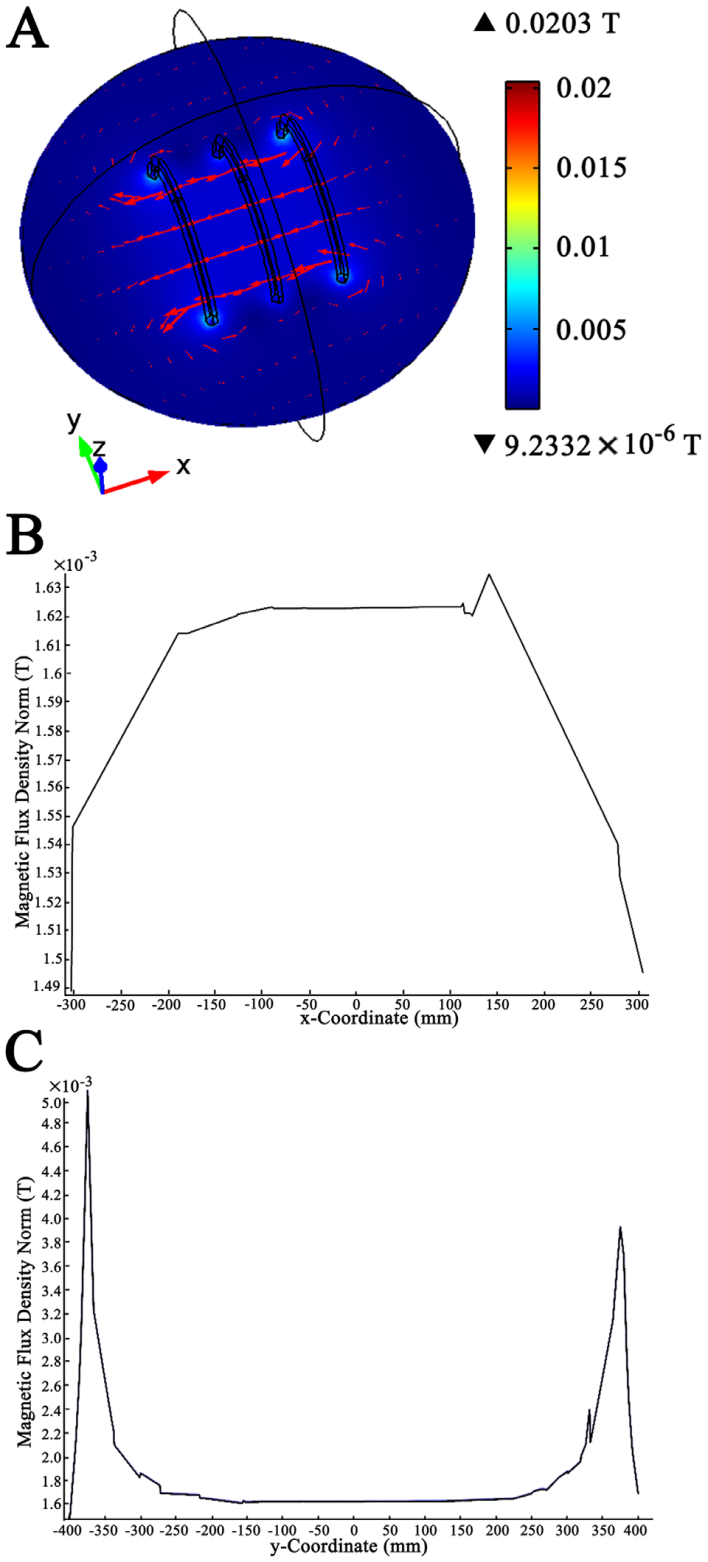
The three dimensional distribution of peak MFD at XY plane (the activity plane of rats) of modified Helmholtz coils and two dimensional distribution of peak MFD on O–X and O–Y cut line of XY plane when the current in the coils reached peak value (approximately 1.5 A). (**A**) Three dimensional distribution of peak MFD at XY plane of modified Helmholtz coils whose homogeneous color (blue) indicates the peak MFD at XY plane was uniform and approximately 1.6×10^−3^ T and red arrows indicates the instantaneous direction of MFD. (**B**) Two dimensional distribution of peak MFD on O–X cut line of XY plane whose major parts at the activity plane of rats was uniform and approximately 1.6×10^−3^ T. (**C**) Two dimensional distribution of peak MFD on O–Y cut line of XY plane whose major parts at the activity plane of rats was uniform and approximately 1.6×10^−3^ T.

A small resistor of 2 Ω was placed in series with the modified Helmholtz coils. The voltage drop across the resistor was observed with an oscilloscope (Agilent 6000 Series, Agilent Technologies, Inc., Santa Clara, CA). Peak value of voltage drop was observed to calculate the peak value of current in the coils so as to obtain the peak value of MFD. In order to make the distribution of MFD in the modified Helmholtz coils more intuitive, the finite element engineering software called COMSOL Multiphysics (v4.3 COMSOL AB, Burlington, MA, USA) was applied to simulate the three dimensional distribution of the MFD in the modified Helmholtz coils when the current in the coils reached peak value (approximately 1.5A). A physics-controlled mesh setting whose element size is coarse was employed to avoid memory overflow caused by too many mesh elements. By establishing the geometric model, setting boundary conditions, meshing models and obtaining numerical solutions, the distributions of MFD of modified Helmholtz coils are shown in Fig. 2. We can find that the MFD on rats’ behavior plane (XY plane) was uniform and the peak MFD was about 1.6×10^−3^ T which approximately coincided with the practical measurement result ((1.6±0.1)×10^−3^ T).

### Evaluation of Mechanical Allodynia

Tactile allodynia was assessed by measuring the hind paw withdrawal threshold to the application of a calibrated series of 6 von Frey filaments (bending forces of 2, 4, 6, 8, 10 and 15 g) (Stoelting, Wood Dale, IL, USA) using a modification of the up-down method [31]. Rats were placed in acrylic cages with a wire grid ﬂoor and allowed to sit in a quiet room for 30 min before beginning the tests. Starting with the filament that has the lowest force (2 g), the filament was applied perpendicularly to the mid-plantar surface of hind paw with sufficient force to cause the filament to buckle slightly. Brisk withdrawal or hind paw flinching was considered as the positive response. Each filament was applied five times to each hind paw (for 6–8 s per stimulation, with a stimulus interval of 1–2 min). Minimum recording of five positive responses (50%) out of 10 stimulations for both paws was considered to be the mechanical withdrawal threshold (MWT) (in grams). Absence of a response (less than five withdrawals) prompted use of the next graded filament. The cut-off of a 15 g filament was selected as the upper limit for testing, since stiffer filaments tended to raise the entire limb rather than to buckle, substantially changing the nature of the stimulus. A significant decrease in the threshold of hind paw withdrawal in response to the mechanical stimulus was interpreted as indicating the presence of mechanical allodynia as compared to the baseline threshold.

### Evaluation of Thermal Hyperalgesia

The thermal stimulation system (Commat, Ankara, Turkey) consisting of a clear plastic chamber (10×20×24 cm^3^) that sits on a clear smooth glass ﬂoor was used to assess thermal hyperalgesia by measuring nociceptive thermal threshold. Before beginning the test, rats were placed individually in the chamber and allowed approximately 30 min to acclimate to the testing environment. A radiant heat source (8 V, 50 W halogen bulb) mounted on a movable holder below a glass pane was positioned to deliver a thermal stimulus to the mid-plantar region of the hind paw. The intensity of the heat stimulus was maintained constant throughout all experiments. When the rat feels pain and withdraws its paw, a photocell detects interruption of a light beam reﬂection, the infrared generator is automatically switched off, and the timer stops, determining the withdrawal threshold. Each rat was unilaterally tested three times at 3-min interval. The average of the three measurements was taken as thermal withdrawal threshold (TWT). In order to avoid excessive suffering of rats, the thermal source was automatically discontinued after 25 s (cut-off latency) if the rat fails to withdraw its paw. A significant decrease in the latency of hind paw withdrawal in response to the noxious thermal stimulus was interpreted as indicating the presence of thermal hyperalgesia as compared to the baseline latency.

### Time Course for Measurements of Weight, Blood Glucose, Mechanical Allodynia and Thermal Hyperalgesia

The weights, blood glucose levels and responses to mechanical and thermal stimuli of all rats were evaluated prior to STZ administration, and there were no statistically significant differences for these parameters among three groups. Weights and blood glucose levels of all rats were regularly measured in the Friday morning (9:00–11:00) in weeks 0, 1, 3, 5 and 7 after PEMF stimulation. Mechanical allodynia and thermal hyperalgesia were evaluated at the Friday night (19:00–23:00) in weeks 0, 1, 3, 5 and 7 after PEMF stimulation. For these two tests, measurements were done by two experimenters who were not aware of the treatment groups respectively and responsible for each test until the study finished.

### Ultrastructural Examination of Sciatic Nerve

Seven weeks after PEMF stimulation in STZ-treated rats with DPN, all rats were anesthetized by an intraperitoneal injection of 7% chloral hydrate solution (0.45 ml/100 g) prior to collecting the sciatic nerve [32]. The distal part of the sciatic nerve was dissected and post fixed by immersion in the fixative solution (2% paraformaldehyde, 2% glutaraldehyde, 0.1 M cacodylate buffer at pH 7.3) for 2 h at 4°C, and washed in 0.1 M cacodylate buffer, and osmicated for 4 h in 1% OsO_4_ (Fluka). Nerves were rinsed in 0.1 M cacodylate buffer, dehydrated and embedded in epoxy 812-Araldite (Polysciences). Ultra-thin sections (80 nm) were subsequently cut, collected on cellodincoated single slot grids and stained with uranyl acetate and lead citrate. Photographs were obtained using a transmission electron microscope (JEM-2000EX, Japan) operated at 80 keV.

### Immunohistochemical Study for VEGF of Sciatic Nerve

The distal part of the sciatic nerve was dissected and post fixed by immersion in the fixative solution (10% paraformaldehyde), and routine paraffin embedding was performed. Longitudinal sections (12 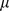
m) of sciatic nerve were thaw-mounted onto Superfrost Plus Slides (VWR). Sections were washed with PBS, and then incubated in blocking buffer (10% normal goat serum, 0.2% Triton-X 100 in PBS) for 1 h at room temperature. Slides were incubated overnight at 4°C with primary antibodies (in blocking buffer): anti–VEGF (1:100; EMD Millipore, USA). Slides were washed and then incubated with goat anti-rabbit IgG (1:200; Jackson ImmunoResearch, USA) for 1 h at room temperature. Digital images were acquired using light microscope (ECLIPSE50i, Nikon, Japan).

### Statistical Analysis

Statistical analyses were carried out using SPSS (version 14.0, SPSS, IL, USA). All values were expressed as means ± standard error of the mean (SEM). P<0.05 was considered statistically significant. Data sets (body weight, blood glucose level and mechanical and thermal withdrawal threshold) of time course study were analyzed by two-way repeated measures analysis of variance (ANOVA). All results were interpreted using the Greenhous–Geisser correction to reduce the probability of obtaining a significant result by chance alone. Between subject factors consisted of intervention (Control, DM and DM+PEMF) and within subject factors consisted of time (weeks 0, 1, 3, 5 and 7 after PEMF stimulation) resulted in a 3×5 ANOVA. Data was analyzed for intervention and time main effects. Bonferroni-adjusted pairwise comparisons were performed for multiple comparisons of the means between the groups. PEMF effect would be indicated by a significant main effect for intervention.

## Results

### Body Weight and Whole Blood Glucose Level

**Figure 3:**
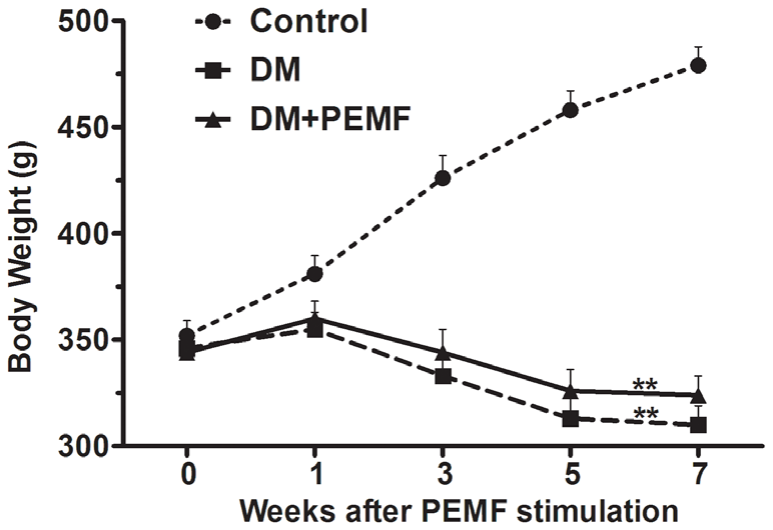
Trends of body weight in Control, DM and DM+PEMF groups in weeks 0, 1, 3, 5 and 7 after PEMF stimulation. Data are presented as means ± SEM for 8 rats in each group. **P<0.01, statistically significant compared to the Control group (Bonferroni-adjusted pairwise comparison regarding the main group effect after two-way repeated measures ANOVA).

Two-way repeated measures ANOVA with a Greenhouse-Geisser correction determined that a significant main effect for time (F (2.109, 75.936) = 9.502, P<0.001) was found for means of body weight throughout the time course. The body weight differed significantly between time points. Post hoc tests using the Bonferroni correction revealed that the mean body weight of DM group and DM+PEMF group were significantly lower than in the Control group (P<0.01) (Fig. 3). After STZ injection, rats consistently lost weight. Although there was slightly less loss of the body weight in PEMF treated diabetic rats, no significant difference between DM+PEMF group and DM group was found (P>0.05). PEMF stimulation did not significantly affect the loss of body weight caused by diabetes.

**Figure 4:**
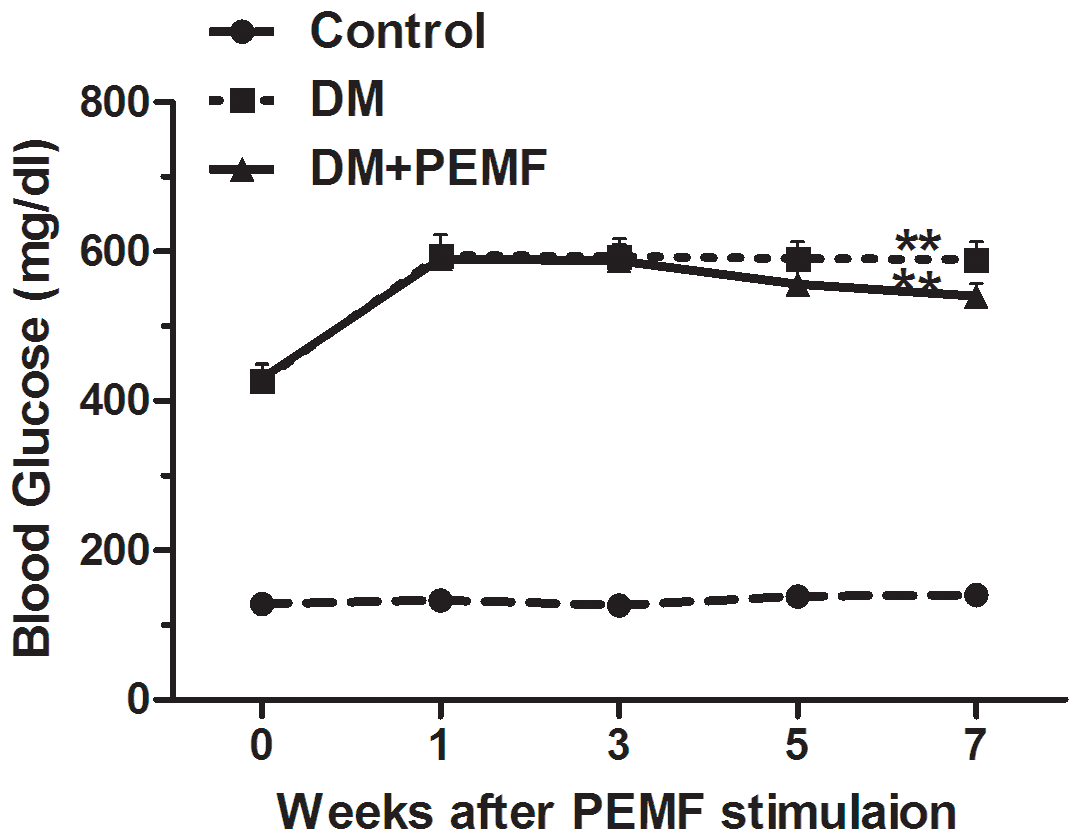
Trends of blood glucose levels in Control, DM and DM+PEMF groups in weeks 0, 1, 3, 5 and 7 after PEMF stimulation. Data are presented as means ± SEM for 8 rats in each group. **P<0.01, statistically significant compared to the Control group (Bonferroni-adjusted pairwise comparison regarding the main group effect after two-way repeated measures ANOVA).

Similarly, a significant main effect for time (F (1.841, 38.651) = 92.331, P<0.001) was observed for average blood glucose level throughout the time course. The average blood glucose level differed significantly between time points. Bonferroni-adjusted pairwise comparisons revealed that the average blood glucose levels of DM group and DM+PEMF group were significantly higher than in the Control group (P<0.01) (Fig. 4). STZ administration caused a rapid elevation of average blood glucose levels (>500 mg/dl) within one week, which persisted for up to 7 weeks. Although the blood glucose levels were slightly lower in PEMF treated diabetic rats, there was no significant difference between DM+PEMF group and DM group (P>0.05). PEMF stimulation did not significantly affect the hyperglycemia caused by diabetes.

### Effects of PEMF on Mechanical Allodynia

**Figure 5:**
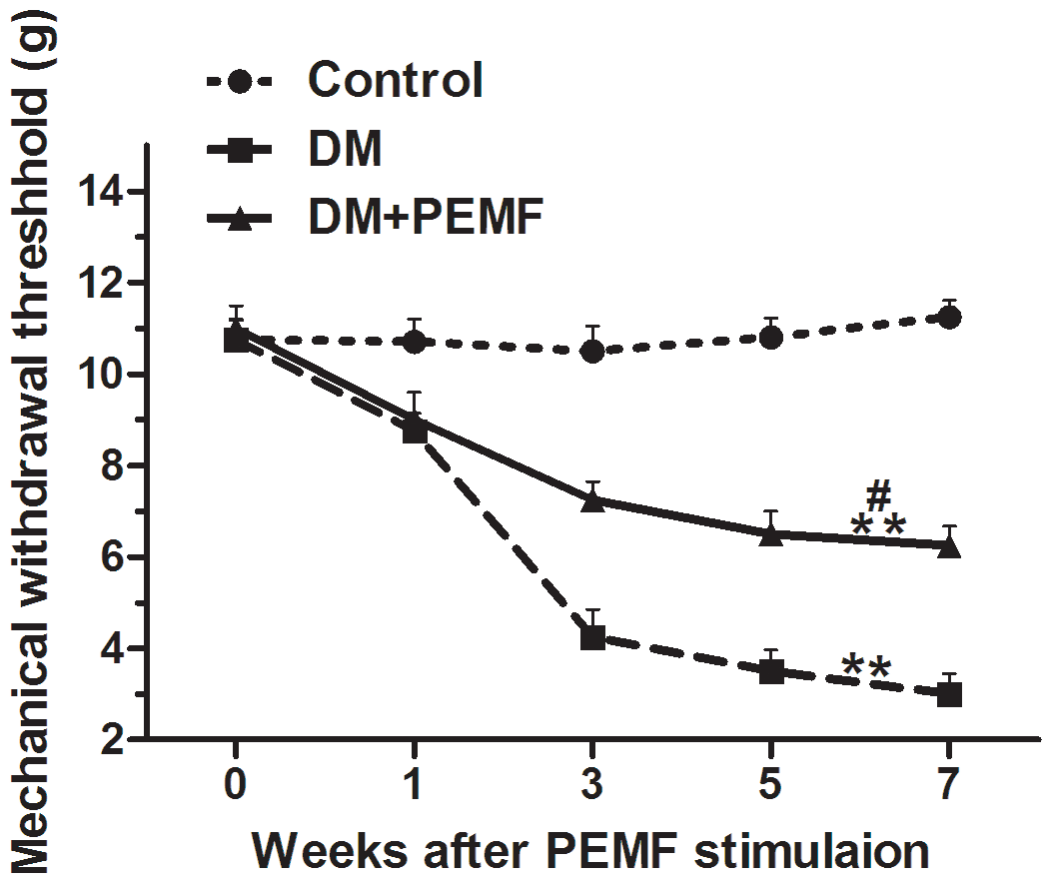
Trends of MWT in Control, DM and DM+PEMF groups in weeks 0, 1, 3, 5 and 7 after PEMF stimulation. Data are presented as means ± SEM for 8 rats in each group. **P<0.01, statistically significant compared to the Control group, ^#^P<0.05, statistically significant compared to the DM group (Bonferroni-adjusted pairwise comparison regarding the main group effect after two-way repeated measures ANOVA).

A significant main effect for time (F (2.975, 62.470) = 176.065, P<0.001) was observed for MWT throughout the time course. MWT differed significantly between time points. Bonferroni-adjusted pairwise comparisons revealed that the MWT of DM group and DM+PEMF group were significantly lower than in the Control group (P<0.01) (Fig. 5). There was a significant difference between DM+PEMF group and DM group (P<0.05) (Fig. 5). PEMF stimulation significantly prevented the development of hypersensitivity to mechanical stimulus in diabetic rats (Fig. 5).

### Effects of PEMF on Thermal Hyperalgesia

**Figure 6:**
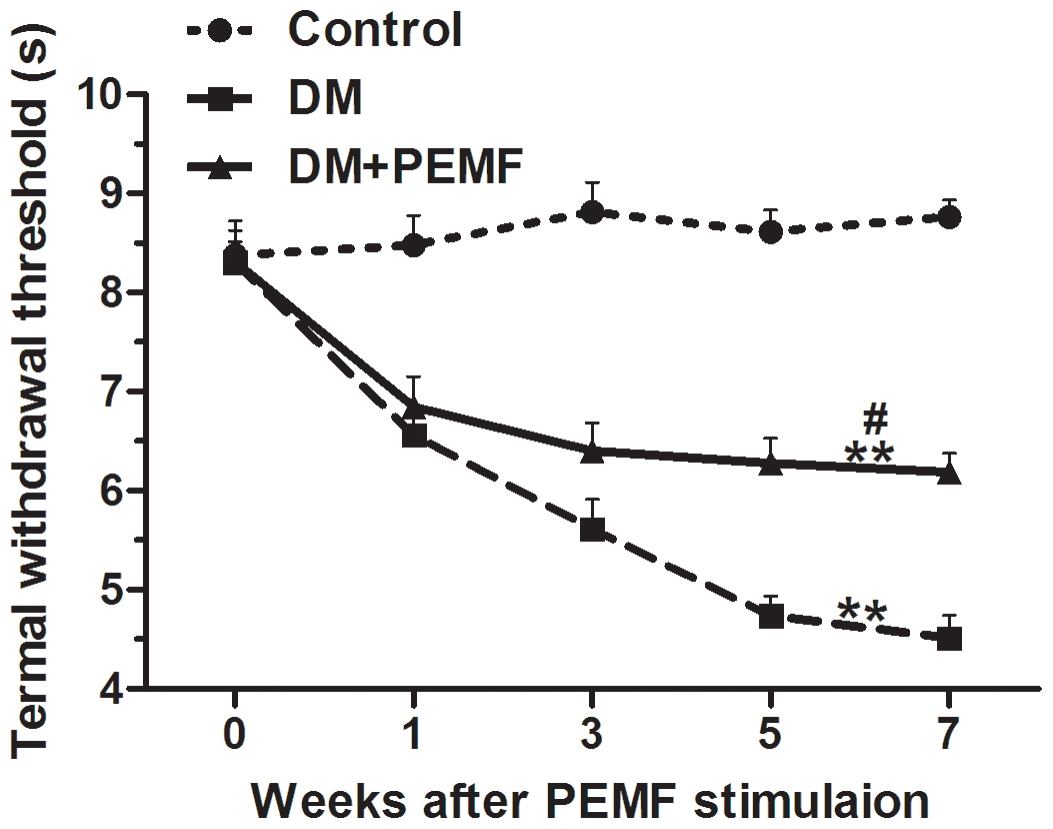
Trends of TWT in Control, DM and DM+PEMF groups in weeks 0, 1, 3, 5 and 7 after PEMF stimulation. Data are presented as means ± SEM for 8 rats in each group. **P<0.01, statistically significant compared to the Control group, ^#^P<0.05, statistically significant compared to the DM group (Bonferroni-adjusted pairwise comparison regarding the main group effect after two-way repeated measures ANOVA).

A significant main effect for time (F (2.564, 53.840) = 56.742, P<0.001) was observed for TWT. TWT differed significantly between time points. Bonferroni-adjusted pairwise comparisons revealed that the TWT of DM group and DM+PEMF group were significantly lower than in the Control group (P<0.01) (Fig. 6). There was a significant difference between DM+PEMF group and DM group (P<0.05) (Fig. 6). PEMF stimulation significantly prevented the development of hypersensitivity to noxious thermal stimulus in diabetic rats (Fig. 6).

### Electron Microscopy of Sciatic Nerve

**Figure 7:**
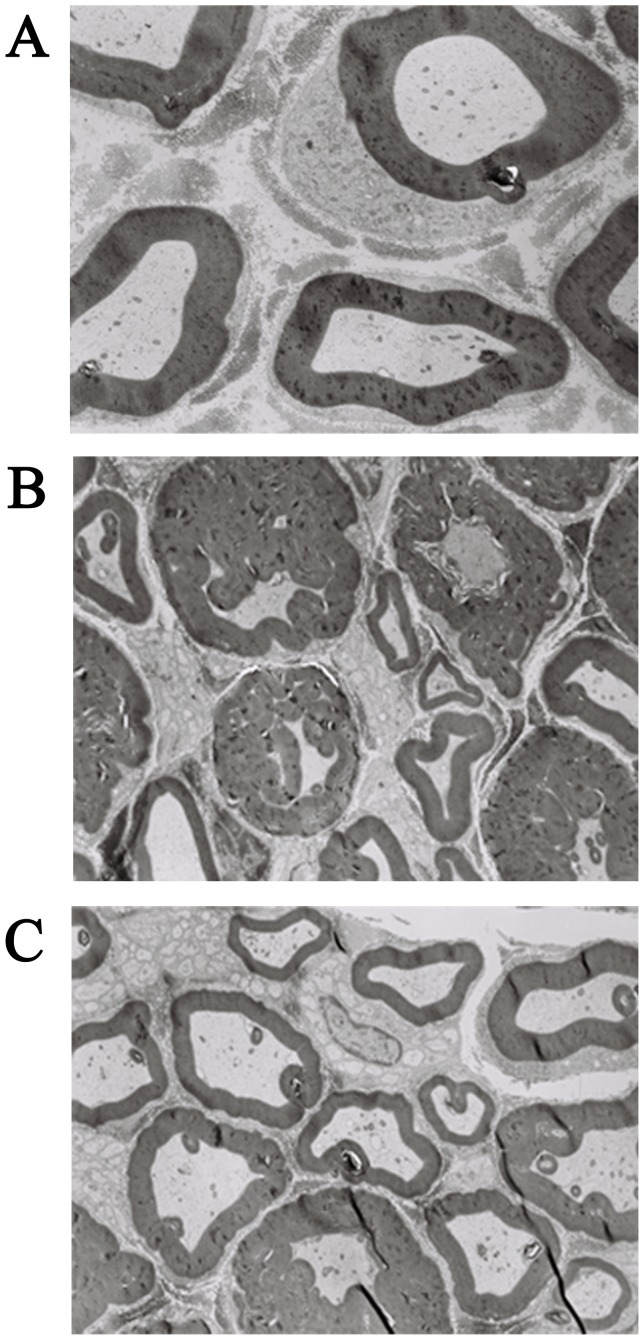
Electron micrographs of sciatic nerves in Control, DM, and DM+PEMF groups after a 7-week experimental period in all rats (magnification: ×6000). (**A**) Control group: Myelinated fiber had normal structure and morphology. Myelin sheath was in integrity and lined up in order. (**B**) DM group: Demyelination and axon enlargement were observed. Myelin sheath showed infolding, splitting, swelling and deformation, and layers were separated or disappeared. (**C**) DM+PEMF group: Myelin sheath of sciatic nerve was abnormal, the densities of layer on myelin sheath were uneven and rarefaction, but the damage was slighter than in the DM group.

Ultrastructural examination of sciatic nerve was obtained by using transmission electron microscopy after a 7-week experimental period in all rats. In Control group, myelinated fiber with normal structure and morphology was observed (Fig. 7A). In DM group, some evidences of axonal degeneration such as demyelination and axon enlargement were observed. Myelin sheath showed infolding, splitting, swelling and deformation, and layers were separated or disappeared (Fig. 7B). In DM+PEMF group, myelin sheath of sciatic nerve was abnormal, the densities of layer on myelin sheath were uneven and rarefaction, but the damage was slighter than in the DM group (Fig. 7C). Seven-week exposure to PEMF stimulation partially prevented the development of axonal degeneration in STZ-treated rats with DPN.

### Immunostaining for VEGF in Sciatic Nerve

**Figure 8:**
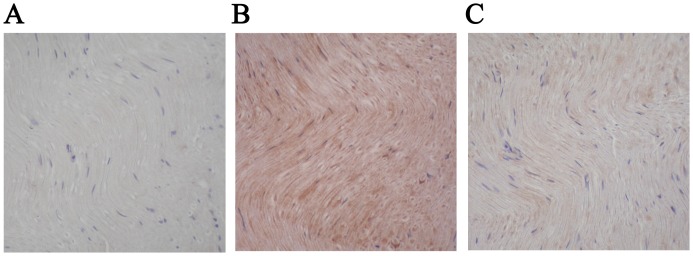
Immunohistochemical staining for VEGF in sciatic nerves in Control, DM and DM+PEMF groups after a 7-week experimental period in all rats (magnification: ×200). (**A**) Control group: No VEGF immunostaining in sciatic nerve. (**B**) DM group: Intense VEGF immunostaining in sciatic nerve. (**C**) DM+PEMF group: Less VEGF immunostaining intensity in sciatic nerve compared to that of the DM group.

After a 7-week experimental period, no VEGF immunostaining in sciatic nerve was seen in Control group (Fig. 8A). In contrast, diabetic rats with DPN showed intense VEGF immunostaining in sciatic nerve (Fig. 8B). Diabetic animals treated with PEMF stimulation showed less VEGF immunostaining intensity in sciatic nerve compared to that of the DM group (Fig. 8C).

## Discussion

Our data in the present study support the hypothesis that PEMF might play a therapeutic role in the development of DPN in STZ-treated rats. Efficacy was evaluated by the assessment of hypersensitivity using behavioral assays of neuropathic pain that included hind paw withdrawal threshold to non-noxious mechanical stimuli (mechanical allodynia) and thermal hind paw withdrawal latency to noxious heat stimuli (thermal hyperalgesia). In our experiments, 3 weeks after the STZ injection, rats with diabetes developed mechanical allodynia and thermal hyperalgesia, which is consistent with previous studies which demonstrated that DPN often comes alone with altered sensitivity by producing both allodynia and hyperalgesia both in STZ-induced diabetic animal models and diabetic patients [33]–[35]. Our results also revealed that application of PEMF attenuated the development of painful DPN. PEMF stimulation showed protective effects to non-noxious mechanical stimuli and noxious heat stimuli and caused an increase in hind paw withdrawal threshold to mechanical stimuli and response time to thermal pain compared to the diabetic rats with sham PEMF stimulation. Our findings are similar to a previous investigation which asserted that treatment with PEMF may prevent the development or may reverse the abnormalities observed in animal models for painful DPN [36]. Although different types of PEMF were employed by these two studies, the same anti-neuropathic pain efficacy emerged.

In the current investigation, a marked decrease in body weight of diabetic rats was observed on week 3 as compared to non-diabetic control rats. The reduction in body weight is probably related to the osmotic diuresis and dehydration induced by diabetic hyperglycemia [36], [37]. Meanwhile, blood glucose level rose immediately after the STZ injection, reached quite a high level at first week, and then remained approximately at a stable value. The results of the current study have confirmed previous findings that blood glucose level is elevated and body weight is decreased in diabetic rats after STZ administration [38], [39]. Our results also revealed that PEMF stimulation did not significantly prevent the weight loss caused by diabetes, which is consistent to a previous investigation [36]. However, contrary to the findings researched by Mert et al. [36], who observed that PEMF had efficacy in anti-hyperglycemia in diabetic rats, we found that the application of PEMF did not significantly alter hyperglycemia in diabetic rats during the whole experimental observation (7 weeks). This finding is consistent with the fact that the hematoxylin and eosin staining for pancreatic islets in diabetic rats with PEMF stimulation and sham PEMF stimulation showed similar atrophy and reduction in cell numbers (not illustrated) in the current study. The different effects of PEMF in hyperglycemia might be ascribed to the different types of PEMF adopted by Mert et al. and us. These inconsistent findings concerning PEMF effects on DPN often come from varying stimulation parameters and exposure durations [40].

Obviously, a prerequisite for a clear understanding of the pathophysiological mechanisms of neuropathic appearance and treatment is to know if there are definite structural changes in the nerve fibers and to what extent they exist in the patients or experimental animal models. The pathology of DPN is characterized by progressive nerve fiber loss [41]. Our morphological analysis was performed on the sciatic nerve because the common type of DPN associated with diabetes in humans is the loss of the distal region of long and large-diameter axons. In the present study, some evidences of axonal degeneration such as demyelination and axon enlargement were observed in STZ-treated rats with DPN. Similar results were also reported by other investigators [42], [43]. The sciatic nerve degeneration associated with morphologic changes was confirmed by hyperalgesia and allodynia in diabetic rats with neuropathy in our study, which is consistent with the findings that pathological changes in diabetic rats with DPN are characteristically associated with altered pain sensitivity [44]. In addition to this, our morphological study of sciatic nerve revealed that long-term PEMF stimulation partially attenuated the development of axonal degeneration observed in STZ-treated rats with DPN, which appears to be seldom reported in animal models for DPN by other investigators.

Our findings demonstrate that diabetic rats with DPN express VEGF in peripheral nerves such as sciatic nerve, while adult and healthy rats did not express the VEGF. Similar findings were also revealed by a previous study [24]. Since it is known that angiogenesis takes primarily place in metabolically altered or in injured peripheral nerves and VEGF has demonstrated neurotrophic functions in both central and peripheral neurons [45]–[47], it is not surprising to find elevated levels of the most potent vascular growth substance in peripheral nerves of diabetic rats with DPN. Intriguingly, on the one hand, direct neuroprotective role for VEGF comes from both in vitro and in vivo studies [8], but on the other hand, a potential consequence of high levels of VEGF observed in diabetes will be enhanced vascular permeability which often results in the extravasation of plasma protein as well as the formation of lesions in peripheral nerves. This abnormal angiogenesis caused by up-regulated VEGF expression initiates chronic insidious progressive damage and loss in unmyelinated and myelinated peripheral nerve fibers [48]. The fact that sciatic nerve of diabetic rats with DPN over seven weeks’ PEMF stimulation showed less VEGF immunostaining might indicate that restitution of nerve function induced by PEMF stimulation leads to down-regulation for VEGF, what’s more, the down-regulated VEGF might in turn cause less damage to peripheral nerve fibers.

This present experimental study demonstrated that treatment with PEMF may attenuate the development of abnormalities observed in animal models for DPN. However, the underlying mechanism of PEMF on DPN is still ambiguous. Previous study reported that PEMF had an significant anti-hyperglycemia efficacy in diabetic rats, and this PEMF-induced reduction in blood glucose level could have a positive effect on nerve function that may result in diminished pain intensity [36]. However, the significant anti-hyperglycemia efficacy of PEMF stimulation was not observed in STZ-treated rats with DPN during the whole experimental observation (7 weeks) in our study. Therefore, we hypothesized that long-term PEMF stimulation would have direct corrective effects on injured nerves, which might lead to diminished pain intensity observed in present studies. Moreover, our hypothesis is supported by in vitro and in vivo studies which have indicated that PEMF stimulation can accelerate nerve conduction velocity and increase compound action potentials of sciatic nerve, enhance nerve growth factor levels, and reduce both oxidative damage and neuronal loss [13], [15], [16].

In summary, the results from our present study demonstrate that treatment with PEMF might prevent the development of abnormalities observed in animal models for DPN. Moreover, it is suggested that PEMF might have direct corrective effects on injured nerves and would be a potentially promising non-invasive therapeutic tool for the treatment of DPN. However, further research is required to elucidate the specific mechanisms of PEMF on DPN and to confirm the applicability of PEMF for clinical practice.
