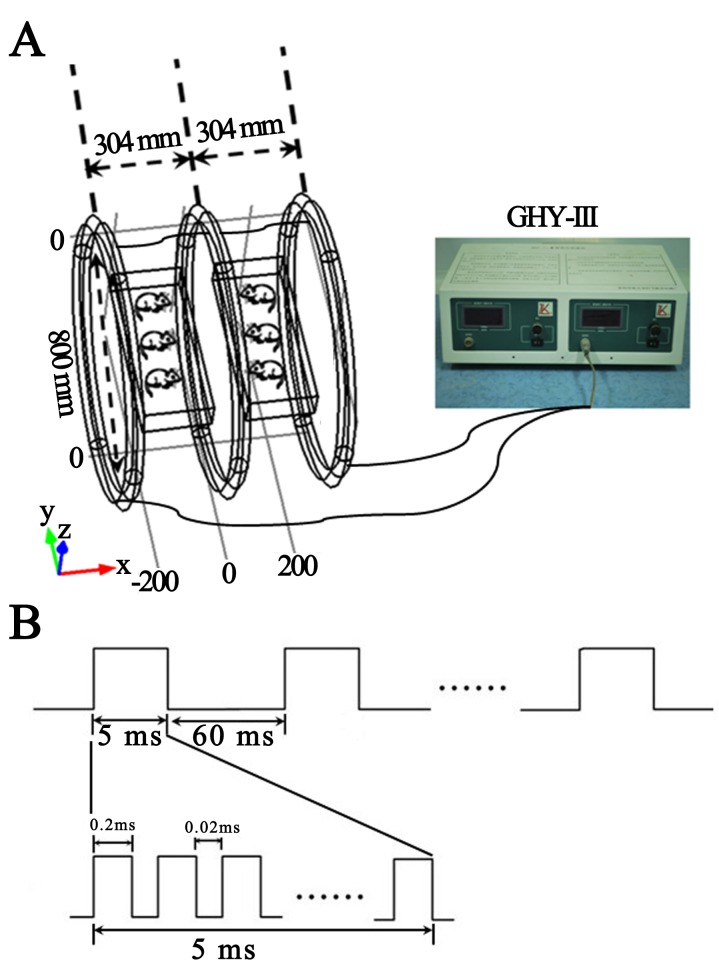# Correction: Therapeutic Effects of 15 Hz Pulsed Electromagnetic Field on Diabetic Peripheral Neuropathy in Streptozotocin-Treated Rats

**DOI:** 10.1371/annotation/b162aa26-6251-4db6-af5f-35b4d9ee5055

**Published:** 2013-12-19

**Authors:** Tao Lei, Da Jing, Kangning Xie, Maogang Jiang, Feijiang Li, Jing Cai, Xiaoming Wu, Chi Tang, Qiaoling Xu, Juan Liu, Wei Guo, Guanghao Shen, Erping Luo

Figure 1 is incorrect. Please view the correct Figure 1 here: 

**Figure:**